# Is the post-multicultural era pro-diversity?

**DOI:** 10.1186/s40878-018-0084-4

**Published:** 2018-05-17

**Authors:** Tamar de Waal

**Affiliations:** Erasmus School of Law, Erasmus University Rotterdam, Burgemeester Oudlaan 50, 3062 PA Rotterdam, The Netherlands

**Keywords:** Post multiculturalism, Interculturalism, Pro-diversity, Civic integration policies

## Abstract

This commentary discusses the claim of Zapata-Barrero that Western countries have historically entered a ‘post multicultural’ phase, in which the emergence of the intercultural policy paradigm must be placed. It argues that this claim is mistaken, or at least too imprecise, and a potential danger for any pro-diversity management strategy, including interculturalism. Moreover, it is highlighted that Zapata-Barrero acknowledges that the contrast between intercultural and multicultural strategies has been overstated in the intercultural literature and that they are in fact complementary. For this reason, instead of reaffirming that interculturalism is temporally post multiculturalism, we should work towards creative synthesis of intercultural and multicultural strategies.

## Introduction

In this commentary, I will discuss the claim of Zapata-Barrero (2017, p. 4) that Western countries have historically entered a ‘post multicultural’ (post-m) phase, in which the emergence of the intercultural policy paradigm must be placed. I believe this claim to be mistaken, or at least too imprecise, and therefore, and crucially, a potential danger for any future pro-diversity management strategy, including interculturalism.

The analysis I put forward rests on the assumption that (liberal) MC and IC strategies as developed in the academic literature are in principle compatible, and, if executed well, can benefit from each other. Considering the articles by Ricard Zapata-Barrero and Tariq Modood in this special issue, this is not a controversial claim. Nonetheless, my main contribution is the premise that if IC advocates suggest that IC strategies, in a temporal sense, come after MC strategies, this strengthens the notion that they are mutually exclusive. This is problematic, as it decreases the chance that IC and MC strategies will be successfully implemented simultaneously in a broader pro-diversity framework. Moreover, it carries the risk of undermining the implementation of pro-diversity strategies (including IC), given that post-m discourses have strongly been about normalizing assimilationist rhetoric and policies. This is a contribution in itself, I believe, but it also sheds light on, first, whether and why MC has been (rhetorically) abandoned, and, second, on how to understand and assess the ongoing burgeoning increase in civic integration policies (‘CIP’) with regard to newcomers in EU Member States.

I will start with a brief review of the relationship between MC and IC. I will then discuss several problems with the statement that we have entered a post-m era, in which the emergence of IC strategies should be understood. Finally, from the perspective of IC strategies themselves, I will list two risks of describing IC as being temporally ‘post’ MC.

## Multiculturalism and interculturalism

Zapata-Barrero and Modood generally agree that MC should not be entirely replaced by IC, but that they complete each other and can be mutually reinforcing. It is obvious that Modood (2017, p. 6) would support this proposition, as he states that IC is best understood as − at certain points − a valuable critique and a version of MC. But also Zapata-Barrero (2017, p. 9) asserts that *‘interculturalism begins then when the multicultural […] policies have developed all their potential, not instead of them, against them or before them […] as complementary paradigms’.*

And indeed, there are many arguments in favor of this position. For one thing, the main goal of both MC and IC is to think broadly through the preconditions for creating a diverse society that is based on individual freedom, equality, and a sense of shared belonging. However, they do not concentrate on the same social entities to achieve this. According to most scholars, MC predominately considers questions related to the ‘multicultural state’ in terms of power sharing and inclusion, while IC focuses more on civil society and the virtues, attitudes, dispositions, and knowledge that individuals need to possess in order to be ‘intercultural citizens’. This means that MC scholars contemplate, for instance, how liberal democracies should accommodate cultural diversity in their public institutions, while IC scholars reflect on how diverse citizens can be stimulated to enter into dialogue and contact in order to increase social bonds. Accordingly, MC and IC strategies predominately lean on different academic bodies of research. Whereas multicultural policy suggestions have been mainly formulated based on theories of justice developed in political philosophy, intercultural policy suggestions regularly refer to studies in social psychology (Levrau & Loobuyck, 2013, p. 7).

For this reason, prima facie, there appears to be no reason to conclude that we *must* choose between either MC or IC strategies. Instead, it seems most fruitful to determine how multicultural state policies can support desired intercultural virtues, and vice versa. Of course, this might not be an easy task. For instance, Modood (2017, p. 6) acknowledges that MC theorists arguably have paid too little attention to how to create institutions to reinforce certain intercultural attitudes on the part of citizens in relation to ‘micro-level interaction’, such as ‘*cultural encounters and everyday interaction in localities, schools, clubs, public spaces’*. However, given that MC and IC are complementary in objective, focus, and methodologies, the ambition to resolve potential tensions between IC and MC strategies is an interesting one, and is not self-contradictory (Kymlicka, 2003, p. 148).

Nevertheless, as I see it, the academic debate about the relationships and tensions between IC and MC have become clouded, because IC theorists most often suggest that IC is fundamentally different and superior to MC, and needs to replace it (e.g., Bouchard, 2011; Cantle, 2016). As previously mentioned, in his contribution to this special issue, Zapata-Barrero’s (2017) thinking on this issue seems to be more nuanced, as he repeatedly states that the new IC paradigm recognizes ‘the strength of the multicultural policy paradigm’ (p. 5). At the same time, however, his analysis remains ambivalent, as he also argues that both the normative and the empirical assumptions underlying MC are outdated (p. 2), or at least have become a lot less relevant (p. 4), or were never proven at all (p. 11). In this context, his statement that the growth of the IC paradigm must be placed in the ‘post-m period’ is most striking. In essence, Zapata-Barrero argues that in light of, among other things, Brexit and the fact that ‘*second-generation migrants are strongly embracing radical outlooks’*, it is evident that ‘*multicultural Europe’* is in a deep crisis, and that IC *‘may help light the way’* (p. 5). All this suggests that the time of MC dominance is over, and we must set foot in a new IC historical era that is more suited to achieving certain societal outcomes (e.g., that support for the EU will trump fears regarding immigration and halt processes of deradicalization).

## Problems with post-multiculturalism

I believe that the aforementioned analysis – presumably intended to demonstrate the relevance and importance of IC – is wrong for a variety of reasons. The first is that, on the level of mere logic, it leaves out several possible hypotheses that would explain certain contemporary developments in Europe that would leave the value of MC strategies untouched. For example, if we accept that MC and IC in principle complete and need each other, it is possible that the problem with the diversity strategies of EU countries has not been that certain MC policies were enacted but that certain IC policies were missing. And if that is true, the solution is not to pursue a post-m framework but to keep MC policies in place and to supplement them with adequate IC strategies. Another possible hypothesis is that we should not overestimate the influence of MC strategies on societal outcomes, in the sense that linking them causally directly to Brexit or to processes of radicalization is unreasonable. MC policies are about creating more fair and inclusive democratic societies, but they are of course not a magic bullet targeted at addressing and resolving all societal issues or ramifications related to diversity in the broadest sense of the term. Furthermore, to suggest that such a direct causal link exists between MC and all sorts of desirable societal outcomes immediately burdens any post-m diversity strategy with an unworkable high standard: namely, the forming of such a magic bullet.

The second reason is that if we approach in a more applied sense the claim that we have entered a post-m era, the relevant questions are whether and how multicultural policies were initially enacted and then abandoned. Surely it cannot be denied that politicians in a remarkable number of European countries explicitly declared that MC belonged to the past. However, it is also well known that, remarkably enough, this also happened in countries – Germany, for instance − that never actively implemented multicultural strategies. Moreover, research suggests that despite the rise of post-m political discourse, the lion’s share of Western democracies since 1980 did not withdraw from MC, but in fact decided to modestly strengthen MC policies (see e.g., Kymlicka, 2012, p. 1; Multiculturalism Policy Index, n.d (MCP Index); Koopmans, Michalowski, & Waibel, 2012). This data evidently blurs the proposition that IC must be academically understood as a response to the end of the MC paradigm, or was born in the context of an era that is truly post-m.

In addition, if we zoom in on particular European countries, it becomes even more evident that the issues involved are more complex than the fact that there was first an MC era and now a post-MC era. To demonstrate this, the Netherlands is a good example, as it is a well-researched country in the international migration literature, and is known for having abandoned its initial multicultural approach to immigrant integration (e.g., Entzinger, 2003, p. 59; Joppke, 2004). And indeed, the MCP Index shows that the Netherlands is one of the few European countries that measurably pulled back from certain MC policies. However, on closer inspection, the idea that the Netherlands, in terms of policies, has ever adopted a full multicultural approach to manage diversity is overly simplistic and untrue. Maarten Vink, for example, speaks of a ‘myth’ that the Netherlands was once a multicultural haven (based on a system of pillarization), as MC was *‘never accepted or practiced as fully and suggested in more stereotypical depictions of Dutch integration politics’* (Vink, 2007, p. 339). The Dutch government did work with notions that could be associated with MC, such as *‘integration with preservation of own identity’,* just after the arrival of post-war immigrants, but this was mostly an *‘excuse for government inaction’* (Vink, 2007, p. 344). Initially, Vink holds that the Dutch integration strategy had little to do with normative MC ambitions, such as aiming at the accommodation of cultural, ethnic, and religious groups in society and group emancipation, but instead was a *‘pragmatic strategy aimed at preparing guest workers to return to their countries of origin, which would be facilitated by integrating foreign workers as little as possible into their temporary host society’* (Vink, 2007, p. 345). In that sense, if the term ‘multicultural’ was used at all in early Dutch integration discourse, it was mostly in a descriptive sense (i.e., that diversity in society was factually increasing), not in the sense that it would be fitting that migrants, under certain conditions, should have a right *‘to express their identities from a assumed symmetry of cultures’* (Vink, 2007, p. 345).

In the same vein, Duyvendak and Scholten argue that MC is not, and never has been, the correct description of the Dutch approach to immigrant integration. Instead, they argue that the term ‘multiculturalism’ to describe the Dutch diversity strategy in the past was ‘coined retrospectively’ (Duyvendak & Scholten, 2011, p. 332). Their findings are very similar to those of Vink; at best, MC has historically been the subject of an influential discussion among other equally significant debates (e.g., liberal-egalitarian and assimilationist) on integration in the Netherlands, but on a policy level the country never structurally and coherently implanted MC strategies on different governmental levels. Moreover, Duyvendak and Scholten assert that this retrospective labeling of policies as ‘multiculturalist’ – both by certain politicians and certain scholars – has to be understood as a ‘performative act’ that served a political agenda (Duyvendak & Scholten, 2011, p. 331). More specifically, their conclusion is that the belief that the Netherlands historically favored multicultural policies was used to serve one purpose: namely, to disqualify the integration and minority policies of the past and to authorize new policies, in particular more assimilationist ones (Duyvendak & Scholten, 2010, p. 40).

## The post-multicultural era as an anti-diversity era

This last point, I believe, adds important information and sheds new light on the MC and IC debate. Of course, the case of the Netherlands is not fully explanatory as regards all the recent rhetorical and political developments in European liberal democracies in relation to MC. However, it does suggest that the discursive rejection of MC in Europe has not genuinely been about fairly discussing the outcomes of certain MC policies, but rather about creating political leeway to further certain assimilationist policy agendas. And if that is true, then this is also bad news for the future of IC strategies, given that MC and IC have, despite their differences, a shared adversity in assimilationist policies. It seems that IC advocates, including Zapata-Barrero, should therefore be careful about uncritically agreeing with − and using − the narrative that Europe has entered a post-m era, as there are signs that this narrative has been mostly about challenging the desirability of respect for certain types of diversity. Put differently, if post-m discussions are indeed aimed at undercutting support for pro-diversity policies, then IC supporters are mistaken, and undermine their own position if they optimistically believe that these discussions inaugurate an IC era.

To illustrate this point, it is insightful to look as well at the stark increase in CIP for newcomers in EU Member States. This development, I believe, must be interpreted as arising against the background of the (anti-diversity) European post-m era. These policies often take the form of language and knowledge-of-country requirements for newly arrived immigrants, and are prerequisites for attaining permanent residency and full citizenship (see e.g., De Waal, 2017; Goodman, 2010; Goodman, 2011; Van Oers, 2013). In the academic literature, several scholars argue that the proliferation of these policies demonstrates that European countries have moved away from MC principles and strategies, and are now opting for more ‘integrationist’ programs (see e.g., Joppke, 2004, 2007). However, also here, this does not seem to be the full story. For one thing, again, most European countries did not actually withdraw their MC policies − if they had them − and CIP strategies therefore have mainly been *combined* with MC strategies (regarding the Flemish ‘combination model’, for instance, see Loobuyck & Jacobs, 2009, 2010). And from an academic perspective, in principle this is no problem at all, as in the same way that MC and IC strategies can be compatible if executed well, CIP and MC strategies can also complement each other (Kymlicka, 2012, pp. 17-18). In brief, from the perspective of MC, CIP policies focusing on, for instance, basic knowledge of the host society’s language(s), history, and institutions, or the importance of the rule of law, can be helpful, as long as they are in accordance with liberal-democratic values, respect cultural pluralism, and are not exclusionary. For this reason, there are no grounds for MC advocates to reject CIP policies *tout court*, for they may support an inclusive society (Levrau & Loobuyck, 2013, p. 12). Along the same lines, there are no academic reasons to conclude that CIP policies are inherently part of societies that are post-m.

Nonetheless, if we look at how CIP strategies are currently enacted in several European states, we can observe two important things: first, despite the potential complementarity of MC and CIP, the new CIP policies have been rhetorically defended as an alternative to MC; and second, they are troublesome from a pro-diversity perspective. Also here, the Netherlands presents a paradigmatic case. Since 1998, the country has changed its CIP policies for newcomers more than 20 times (see e.g., Besselsen & de Hart, 2014, pp. 15-25). And these policies gradually shifted from emancipating and improving the position of certain immigrant groups addressed as a public responsibility to forming a selective political tool to measure the eligibility of individual immigrants to be deemed ‘worthy’ of obtaining secure residency rights or citizenship. For that reason, I demonstrated in earlier research that the CIP strategies of the Netherlands, together with those of other EU Member States, have increasingly counterproductive societal outcomes, and jeopardize the democratic principle of equal citizenship (De Waal, 2017).

As I see it, this recent history of CIP policies in Europe illustrates that IC advocates should be careful in supporting the proposition that Europe is entering, or should enter, a post-m era. The discursive rejection of MC in Europe has not been part of a benign political search to find the best public policies to manage diversity in an inclusive society. Instead, the rise of exclusionary and assimilationist CIP policies indicate that the Europe that has declared itself to be post-m is, above all, less willing to genuinely further public strategies that are pro-diversity.

## MC from the perspective of IC

So what does this tell us about the relationship between MC and IC? I think it is clear that a plea in favor of IC does not benefit from framing it explicitly as existing in, or requiring, a post-m context. We have seen that the empirical foundations of this analysis – in Europe but in North America and Australia as well – are shaky (Kymlicka, 2012). Furthermore, I hope to have demonstrated convincingly that, in a more political sense, the post-m era in Europe has not formed a fertile set of circumstances in which to effectively implement pro-diversity strategies. Therefore, if we look at the ambitions of Zapata-Barrero (2017), who wants the *‘main pillars of MCP […to…] remain’* (p. 4) while he also pleads in favor of an additional ‘*contact-based policy approach’* (p. 2), it is surprising that he decides to reaffirm the end of the MC era. More to the point, if Zapata-Barrero accepts that the IC and the MC contrast has been overstated in the IC literature, and exacerbates the anti-multicultural backlash, should he then not rethink his claim that we are in and need a post-m era? If there is no cleavage between IC and MC, should we then not work towards a creative *synthesis* of IC and MC strategies, instead of implying that we should accept that we are in a post-m era? I believe he does, because any defense of IC that contrasts IC and MC, including those that claim that IC is temporally post MC, runs into two main problems that might eventually jeopardize the ambitions of IC strategies themselves.

First, it is unwise for IC supporters to discard − or not stress strongly − the previously mentioned complementarily of IC and MC, because the strategies are mutually reinforcing. To be more specific, this means that if a state is not MC, and, for example, mistreats immigrant minorities by not promoting a national identity that is inclusive with regard to immigrant groups, successful intercultural contact between diverse citizens in terms of equality also becomes highly unlikely. Zapata-Barrero does acknowledge this occasionally (e.g. Zapata-Barrero, 2017, p. 9), but still presents the emergence of IC as being attached to the post-MC era and not so much to MC. However, this ambivalence makes it unlikely that states that are sympathetic toward pro-diversity policies will − on the basis of the author’s work − implement adequate MC policies together with IC strategies. What would be their motivation to do so if MC is generally outdated and has failed? This is troublesome, because if certain MC strategies are indeed a crucial intergradient to make IC strategies work, IC policies that are too weakly embedded in a MC state will ultimately also be qualified as a failure, and cannot, in themselves, guarantee a fair society. For this reason, IC advocates should unequivocally argue that pro-diversity policy frameworks are based on a right combination of MC and IC (alongside, I would say, CIP) policies, adjusted to the specific historical contexts of countries. To find such a fitting combination might not be easy, but crafting the best public policies never is.

Second, when IC supporters join in post-m rhetoric, they might underestimate its anti-diversity origins. And potentially, this makes implementing pro-diversity strategies in Europe even more difficult than ever, as it fuels a societal ethos in which assimilation is normalized. For that reason, as a final note, it is worth highlighting that finding the most appropriate response to the rhetorical abandonment of MC in Europe forms a major challenge for pro-diversity advocates. On the one hand, Kymlicka might be right in that the rhetorical retreat from the word ‘multiculturalism’ could be helpful for pro-diversity agendas if it mainly *repackages* the labels used for progressive diversity strategies, given that the term MC has fallen out of favor in certain European countries (Kymlicka, 2016). In other words, pro-diversity advocates could be interested in rebranding MC while remaining equally committed to its underlying normative principles, because they believe the term is politically beyond recovery, and that a new term will be more effective. Perhaps this is how Zapata-Barrero understands his IC framework. On the other hand, however, if pro-diversity advocates decide to passively permit or even approve the consensus that MC is ‘behind us’, they also counterproductively legitimize political outlooks and create momentum for policy proposals that are fundamentally anti-diversity.
